# Aetiology, Diagnosis and Treatment of Arterial Occlusions of the Retina—A Narrative Review

**DOI:** 10.3390/medicina60040526

**Published:** 2024-03-23

**Authors:** Barbara Daxer, Wolfgang Radner, Florian Fischer, Andreea-Liliana Cocoșilă, Armin Ettl

**Affiliations:** 1 Karl Landsteiner University of Health Sciences, Dr. Karl-Dorrek-Straße 30, 3500 Krems, Austria; 2 Department of Ophthalmology and Orbital Surgery, University Hospital St. Pölten, Dunantplatz 1, 3100 St. Pölten, Austria; 3 Austrian Academy of Ophthalmology, Mollgasse 11, 1180 Vienna, Austria; 4 Faculty of Medicine and Pharmacy, University of Oradea, 1 Decembrie Square 10, 410068 Oradea, Romania

**Keywords:** retinal artery occlusion, central retinal artery occlusion, arterial branch occlusion, vascular risk factors, retinal vascular disease

## Abstract

Arterial occlusions of the retina are potentially sight-threatening diseases which often result in profound visual loss. The aim of this narrative review is to provide an overview of the aetiology, discuss major risk factors, describe the management and systemic assessments and evaluate existing therapies. For this review, an extensive literature search in PubMed was performed. Emboli from the heart or the carotid arteries can cause ophthalmic artery occlusion (OAO), central retinal artery occlusion (CRAO) and branch retinal artery occlusion (BRAO). Most patients with arterial occlusions have vascular risk factors such as arterial hypertension, hyperhomocysteinaemia, carotid stenosis and atrial fibrillation, which also increase the risk of cerebral stroke and myocardial infarction. Therapies such as ocular massage, thrombolysis and anterior chamber paracentesis have been suggested but are still equivocal. However, it is evident that retinal artery occlusion should be immediately treated and accompanied by interdisciplinary collaboration, since early diagnosis and the proper treatment of possible risk factors are important to reduce the risk of further damage, recurrences, other vascular diseases and mortality.

## 1. Introduction

The purpose of this review article about arterial occlusions of the retina is to provide an overview of their aetiology, major risk factors, medical management and systemic assessments, and to evaluate existing therapies.

Acute ophthalmic artery occlusions (OAO) or retinal artery occlusions (RAO) of the central retinal artery (CRAO) or branch retinal arteries (BRAO) can cause sudden visual loss in the affected eye [1,2,3]. The extent of the visual loss depends on the size of the occluded vessel, the duration of the occlusion and the location of the artery involved.

The first branch of the ophthalmic artery gives rise to the central retinal artery, and also supplies the anterior segment through the ciliary arteries via the rectus muscles. The ciliary arteries, in turn, supply the outer retina via the choriocapillaris, and the central retinal artery supplies the inner retina [1]. Since the ophthalmic artery is the first branch of the internal carotid artery, emboli from the heart or from the two carotid arteries have a direct route to the blood supply of the eye.

The branches of the ophthalmic artery are the arteria centralis retinae (the first branch), the arteriae ciliares posterior longae, the artieriae ciliares posterior breves, the arteria lacrimalis, the arteria supraorbitalis, the arteriae ethmoidalis anterior and posterior, the arteriae palpebrales mediales, the arteria dorsalis nasi and the arteria supratrochlearis. The main vessel supplying the retina is the central retinal artery, which enters the eyeball together with the optic nerve and divides at the optic nerve disk into four main retinal branches. The paired arteriae ciliares posteriores longae, which also arise from the arteria ophthalmica, supply the anterior segments of the eye and the choroid. They follow the course of the optic nerve and laterally pierce the sclera. Within the sclera, they run to the front of the eye, branch to the corpus ciliare and end in an arterial vascular network of the iris (circulus arteriosus iridis major). The arteria ophthalmica also gives rise to around 10–15 branches called arteriae ciliares posteriores breves, which also follow the course of the optic nerve and move with it into the eyeball. There, they form the arterial vascular network of the choroid. The terminal branches of the arteriae ciliares posteriors breves also end in the circulus arteriosus iridis major. In addition, muscular branches of the arteria ophthalmica and the arteria lacrimalis are also involved in the blood supply of the ciliary plexus [4,5].

The prognosis of OAO is poor, because this occlusion blocks the blood supply to the retina and to the choroid. In most cases, OAO results in profound vision loss (hand motion, light perception or even worse) and is likely to be accompanied by ischemic neovascularisation such as rubeosis [3]. OAO usually causes an extensive and more pronounced ischemic whitening of the retina than CRAO, and usually does not cause a cherry red spot because of the reduced perfusion of the choroid [6].

CRAO also results in profound visual loss. Visual acuity is at risk of deteriorating to 20/200 Snellen (0.1 decimal), counting fingers or even worse. As in OAO, an ipsilateral relative afferent pupillary defect may be detectable [3,7]. In complete CRAO, permanent retinal damage occurs within 90 min [7]. In cases of incomplete CRAO, vision may return after a delay of 8–24 h [6]. Initially, the retina might appear normal, but then an ischemic thickening of the nerve fiber layer leads to a whitening of the macula, and a typical cherry red spot can be seen in the fovea because the blood supply of the choroid is still present.

BRAO has a better visual prognosis and is caused by smaller emboli. In many cases, patients with BRAO experience an inferior or superior shade. The extent of the visual loss also depends on the location of the occlusion [3,8]. The more central the occlusion, the worse the resulting visual acuity. The affected retinal sector looks greyish and pale. In up to 68% of cases, a visible embolus can be found [6].

In about 32% of eyes, a cilioretinal artery exists, which arises from the short posterior ciliary arteries. In fluorescein angiography (FFA), the cilioretinal artery fills just before the central retinal artery [6]. Because the cilioretinal artery originates from the chorioretinal circulation, patients suffering from CRAO benefit from this collateral macular circulation and have a better visual prognosis [3,6,7]. However, the cilioretinal artery can also become occluded, leading to significant visual impairment. 

Three distinct patterns of cilioretinal occlusions (CLRAO) have been described: (a) isolated occlusion (isolated CLRAO) is often associated with atherosclerosis of the carotid arteries. The visual loss is moderate in most cases (90% achieve 0.5 decimal); (b) CLRAO can also be combined with a central venous occlusion (CRVO). This occurrence can be explained by several mechanisms, such as an increase in pressure in the cilioretinal artery secondary to the increased capillary pressure caused by CRVO, by a simultaneous reduction in the perfusion pressure of the cilioretinal arteries and retinal veins or by a vasculitic process in the optic nerve; (c) CLRAO can also be combined with anterior ischemic optic nerve neuropathy. The blood supply of optic discs arises from the posterior cilioretinal arteries, from which the cilioretinal artery also originates. [1,6,8]. Since both the optic nerve head and the cilioretinal arteries are supplied by the posterior cilioretinal arteries, the concomitant occurrence of an anterior ischemic optic neuropathy with a CLRAO is very rare, and is mostly caused by arteritic diseases. Fundoscopically, an optic disc-swelling and flame-shaped hemorrhages occur in the superior nerve fiber area, and a well-marked retinal ischemia superior to the fovea can be seen in the affected eye [8]. The visual prognosis is very poor (usually less than 0.05 decimal) [6]. 

Combined retinal artery and retinal vein occlusions are rare events, accounting for only 0.3% of all retinal vascular occlusions in a three-year study by Ravel et al. [9]. Nevertheless, retinal vein and artery occlusions can occur in any combination [9]. Most patients present with CRAO combined with a central retinal vein occlusion (CRVO); CLRAO combined with BRVO appears only occasionally, and the least frequent combination is BRVO with BRAO. Various pathological mechanisms have been suggested to cause the combined occlusion of retinal arteries and veins. A sudden increase in retinal intraluminal capillary pressure secondary to CRVO can lead to CLRAO. Similarly, a reduced perfusion pressure of the cilioretinal and retinal arteries reduces the overall retinal circulation and can lead to venous congestion and thrombosis. In general, the prognosis of patients with combined vascular occlusion is poor. Most of the affected eyes develop severe complications, such as rubeosis iridis and neovascular glaucoma [9].

## 2. Aetiology

Prevalence: OAO is very rare and increases with age. The estimated annual incidence of acute RAO ranges from 0.85 to 2 cases per 100,000 persons [3,6,10]; that of CRAO is approximately 1 case per 100,000 people, less than 2% of whom present with bilateral CRAO [7].

Pathomechanism: Emboli are the most common aetiology associated with RAO [1,11]. There are three types of emboli involved in RAO: (a) cholesterol emboli, which appear with a yellow to white color (Hollenhorst plaques) [1,12]; (b) fibrin platelet emboli, which appear more grayish; and, in rare cases, (c) other materials like fat or vegetation from bacterial endocarditis [1]. The most common origin of emboli associated with RAO is suggested to be the atheromatous plaques of the carotid arteries. Severe atheromatous plaques of the internal carotid artery can be found in up to 40% of patients with acute RAO [3]. Recent studies also indicate that a considerable number of emboli arise from cardiac sources such as atrial fibrillation, and aortic and mitral valve diseases [13]. Accordingly, Mac Grory et al. found, in a retrospective study in patients with CRAO, a two years’ cumulative incidence of atrial fibrillations of 49.6% [14].

Risk factors: It has to be noted that most patients with RAO show a co-morbidity of vascular risk factors, such as diabetes mellitus, dyslipidemia, hypertension, hypertensive crises and/or acute coronary syndrome, at the time of RAO [3]. Callizo et al. found undiagnosed vascular risk factors in 78% of patients with CRAO [15]. Most of the patients exhibited carotid artery stenosis, arterial hypertension, and cardiac diseases such as coronary artery disease, atrial fibrillation and valvular heart disease [15]. In addition, in a minority of cases, vasospasm, internal carotid artery dissection [16] and systemic hypotension have also been suspected to be responsible for RAOs [1], and Hayreh et al. found a patent foramen ovale in 2% of their CRAO patients [10]. In a study by Chen et al., over 90% of cases with multiple emboli were highly associated with severe ipsilateral carotid occlusion, exhibiting a considerably decreased hemodynamic flow; in 40% of cases, there was total stenosis of the carotid artery [17].

Less common, but noticeable, are occlusions due to inflammation in or around the vessel wall of the arteries (about 1–5% of all cases) [2]. Such occlusions are mostly associated with giant cell arteritis [2]. Other occlusions originating from vascular inflammations are rare, and can be caused by systemic lupus erythematosus, Wegener granulomatosis, Behcet’s disease, dermatomyositis and polyarteritis nodosa [1,6]. A possible association with anaemia [18] and hyperhomocysteinaemia should also be taken into account [19,20,21], as plasma homocysteine levels of 4–5 mmol/L increase cardiovascular risk to between 32% and 60% [22], and thus also increase the risk for RAOs. Furthermore, infectious diseases such as toxoplasmosis, acute retinal necrosis, cat scratch disease and loiasis can cause inflammation in the retina, and lead to occlusions of adjacent retinal arteries [6]. 

In addition, congenital or acquired thrombophilia increases the risk for vascular occlusions. Examples for congenital thrombophilia include the factor-V-Leiden mutation, protein C deficiency, protein S deficiency and antithrombin III deficiency. Acquired thrombophilia can be caused by antiphospholipid syndrome, hyperhomocysteinemia, myeloproliferative disorders, pregnancy, oral contraceptives or hormonal therapy [4].

CRAO has also been reported in association with structural abnormalities of the eye such as optic disc drusen and pre-papillary loops [6,23,24,25]. Traumatic injury to the orbit and its contents is another risk factor that can result in arterial occlusions, which are sometimes accompanied by retrobulbar hemorrhages. Retinal artery occlusions have also been found in younger patients with vascular spasms caused by migraines or cocaine use [6]. In addition, intense physical exercise leading to dehydration represents a further risk factor for vascular occlusions [1].

Regarding the COVID-19 crisis, Yeo et al. [26] found an association between retinal vascular occlusions and COVID-19 infection in patients at risk of systemic hypercoagulability and thromboembolism. A significant surge in mucormycosis was reported in the Indian subcontinent during the second wave of the COVID-19 pandemic, and CRAO in was aggressive COVID-associated mucormycosis patients, quickly resulting in blindness [27]. Of further concern regarding COVID-19 is a controversial relationship between COVID-19 vaccination and retinal occlusion, with several studies and case reports documenting retinal vascular occlusions following COVID-19 vaccination [26,28,29,30,31,32,33]. Accordingly, Li et al. reported a significantly increased risk of retinal vascular occlusions 2 years following COVID-19-vaccination [34]. A retrospective analysis about the onset-to-arrival time of acute CRAO patients indicated that hospital presentation was delayed during the COVID-19 crisis [35]. 

CRAO can also be induced iatrogenically (a) through retrobulbar anesthesia (most of these patients had vascular risk factors) [36,37,38] and (b) after treatment with cosmetic facial filler injections in the glabellar or nasal region, which were accidentally injected into the facial artery [39]. Autologous fat is the most common cosmetic facial filler associated with severe visual loss [40,41,42].

Occlusions of retinal arterioles can occur after severe head trauma, compressive chest injury or vascular disorders, resulting in a characteristic clinical phenotype called Purtscher’s retinopathy (the occlusion of small retinal arterioles by microemboli) [6]. This type of occlusion is funduscopically characterized by large patches of retinal whitening, cotton wool spots and intraretinal haemorrhages at the posterior pole; optic disc swelling may also occur. The condition is usually bilateral, but unilateral cases have also been reported [6].

With respect to the pathophysiology of RAOs, vascular autoregulation and cytotoxic effects mediated by nitric oxide or glutamate should also be considered. The autoregulation of the arterial pressure within the retina protectively stabilizes the retinal blood flow against local changes, fluctuations in the systemic arterial blood pressure and intraocular pressure. This is suggested to be mainly mediated by the myogenic response of small arteries and arterioles, which constrict when the arterial pressure increases and dilate when it decreases [43]. Autoregulation mediated by nitric oxide (NO) plays an important role in the pathophysiology of neuronal injuries during hypoxia–ischemia [44]. The release of NO induces vasodilation in order to maintain the retinal perfusion, and thus represents a protective response under hypoxic/ischemic conditions [44]. Accordingly, Yilmaz et al. obtains significantly increased concentrations of NO in the aqueous humor of eyes with CRAO [45]. In addition, the vascular endothelial growth factor (VEGF) stimulates the production of NO, and a possible negative effect of NO has been suggested in cases of retinal artery occlusions following intraocular injections of anti-VEGF. The inhibition of VEGF by anti-VEGF injections is hypothesised to reduce the synthesis of NO, which may increase the predominance of vasoconstrictive effects, leading to increased peripheral resistance. From cancer therapy, it is known that anti-VEGF increases the risk of arterial thromboembolism [46,47,48]. Although the reason for this side effect is unknown, the increased risk of arterial thromboembolism and the unfavorable effects of VEGF inhibition should be taken into consideration when treatment with a VEGF-inhibitor is indicated, particularly in retinal diseases, which are primarily ischemic [46].

## 3. Diagnosis

Funduscopy is crucial for the diagnosis of RAO. Although the retina might initially appear fairly normal, the retinal ischemia leads to edema, which appears as ischemic whitening, particularly when the macula is involved [6].

Sudden and painless vision loss is typical for non-arteritic CRAO, while, in arteritic CRAO, headache and a claudiacatio of the jaw can be present [1,3]. The typical sudden visual loss can occur at any time of the day [11]. Hayreh found that vision loss noted when the patients woke up in the morning was associated in 35% of patients with non-arteritic-CRAO, in 29% with non-arteritic-CRAO with cilioretinal artery sparing, in 39% in transient CRAO and in 30% with arteritic CRAO. The author concluded that this is likely to be caused by embolism, thrombosis or a transient CRAO due to a decrease in perfusion pressure during nocturnal arterial hypotension [11].

**Figure 1:**
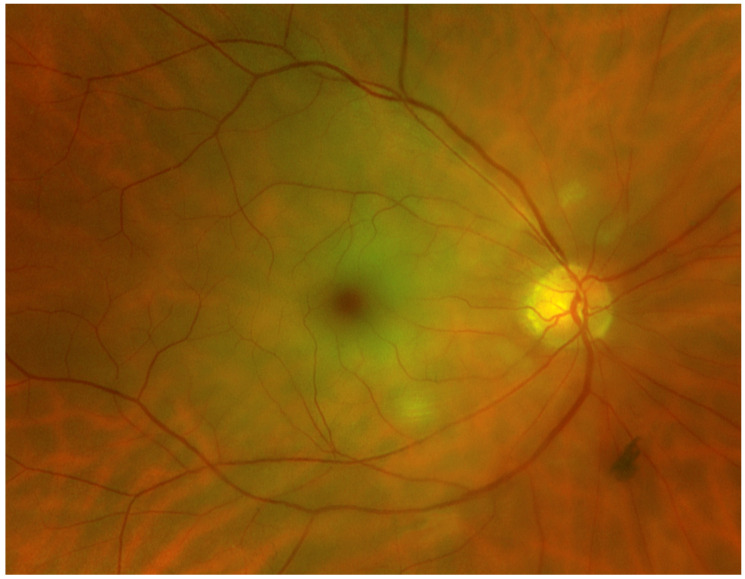
Fundus photograph of a right eye showing a CRAO with diffuse retinal whitening and a cherry red spot in the fovea. (Fundus photograph taken with Optos Panoramic Ophthalmoscope–P200DTx, Optos GmBh, Düsseldorf, Germany).

Acute CRAO can cause a cherry red central spot, which results from the preserved choroidal circulation visible beneath the fovea [6] (Figure 1). In both CRAO and BRAO, an attenuation or segmentation of the retinal arteries may be seen. Breaks in the blood column of the arteries and arterioles, also called “box-carring”, are a further visible sign of RAOs (except in cases of transient CRAO). In some cases of BRAO, the emboli are visible [6].

In the chronic stage of CRAO, a homogeneous scar replaces the inner layer of the retina. Several weeks after acute CRAO, the retinal edema resolves, the opacity disappears, and the retina remains thin and atrophic. In addition, a narrowing of the arteries and atrophy of the optic disc may occur. In some cases, reperfusion may appear. Although the retina seems to appear deceptively normal, a rubeosis iridis can be found in up to 18% of cases [6].

The optical coherence tomography (OCT) images show distinct patterns in the acute and chronic phase of the occlusions. In the acute phase of CRAO, there is an increase in the reflectivity and thickness of the inner retina, and a corresponding decrease in reflectivity in the outer layer of the retina. In follow-up OCT images (chronic phase), there is decreased reflectivity and thickness of the inner retinal layers, and increased reflectivity in the outer retina. In the late stages of CRAO, the retina shows a generalized atrophy of the neurosensory retina, a marked retinal thinning with loss of stratification of the inner retinal layers and a loss of the normal foveal depression [49,50,51]. 

Images from OCT can also show the pathology of paracentral acute middle maculopathy (PAMM), which occurs in patients with retinal capillary ischemia; the OCT images show the subtle parafoveal lesions deep in the retina. [52]. In a retrospective study, Liang et al. [41] found PAMM in 63% of patients with CRAO and in 36% of patients with BRAO. Interestingly, the follow-up examinations revealed that the visual outcome was better in patients with PAMM, than in those without PAMM [52]. 

OCT-angiography (OCT-A) is a diagnostic approach that provides important information about the vascular perfusion of the retina. It allows for the detection of non-perfused areas of the retina in patients with acute and chronic RAO, which are located in the superficial and deep capillary plexuses [53].

Although conventional FFA is usually not an essential examination in the acute phase of a funduscopically verified CRAO, it is helpful for diagnosing small BRAOs. In CRAO, there is delayed perfusion and an absence or stasis of the retinal arterial circulation [3,13], while the choroid fills normally [6]. In contrast, in BRAO, fluorescein cannot be found distal from the occlusion, although some retrograde filling of the occluded vessel from surrounding vessels can appear [6]. 

Electroretinograms (ERG) of eyes with CRAO show an intact a-wave (derived from the photoreceptors) and a reduction in or loss of the b-wave (from Müller and bipolar cells). In cases of BRAO, similar abnormalities can be found by means of multifocal ERG in the affected area of the retina. In OAO, both the a- and b-waves are absent [6].

Visual field testing has rarely been investigated in research studies of patients suffering from CRAO, and few studies have reported preservation of temporal peripheral vision. In BRAO, an altitudinal visual field defect can usually be found, which corresponds to the affected area of the retina [6].

## 4. Systemic Assessment and Management of Retinal Artery Occlusion

Patients with acute visual loss need to have an immediate same-day examination by an ophthalmologist to confirm the diagnosis of CRAO or BRAO and to rule out non-vascular ocular problems such as vitreous or chorioretinal hemorrhage, retinal detachment or acute optic neuropathy, which can also cause sudden visual loss [2,3,54]. Examinations such as checking the pulse, measuring blood pressure and cardiac auscultation may already be used to detect internal pathologies associated with RAO. It is also recommended that blood tests be conducted for thrombophilia screening, thyroid function, rheumatoid factor, anticardiolipin antibody, antinuclear antibody, and glucose, lipids and homocysteine (glucose and lipids should be measured in fasting blood). Urea and electrolyte values might provide information about possible dehydration [1].

Emboli: Since the main source of retinal emboli are atheromatous plaques of the internal carotid arteries, a duplex ultrasound investigation of the carotid and cervical arteries has to be initiated [1]. In addition, further medical imaging, such as computed tomography (CT), magnetic resonance angiography or transthoracic echocardiography, can also help to better specify the embolic sources, including cardiac valve abnormalities [2,55,56,57]. In 52% of patients with RAO, Hayreh et al. [13] found that cardiac echographs revealed an embolic source: 26% came from the mitral valve, 38% from the aortic valve, and 36% from both mitral and aortic valves (mainly valve calcification and prolapses). In 2%, a patent foramen ovale could be identified [13].

Another important factor for RAO is cardiac arrhythmia, particularly atrial fibrillation that increases the risk of thrombus formation in the left atrial appendage [55,56,57]. Paroxysmal atrial fibrillation and intermittent arrhythmia can be detected by long-term ECG (Holter ECG) [56,57]. In the case of atrial fibrillation, therapy including anticoagulation can be considered [2].

Arteritic origin: Up to 5% of CRAOs have an arteritic origin, mostly caused by giant cell arteritis (GCA). Such cases need to be treated with high-dose corticosteroids as soon as possible to protect the fellow eye [1,3]. It is therefore recommended to initially exclude an arteritic origin in patients over 50 years of age [2,58]. Together with an urgent blood test (full blood count, CRP and erythrocyte sedimentation rate) [1,3,59], other typical symptoms of GCA, such as headache, scalp tenderness, jaw claudication, weight loss and existing polymyalgia rheumatica, can be helpful indicators [3]. These symptoms only occur in patients with RAO of arteritic origin. RAO caused by emboli usually presents as a painless, sudden vision loss [1].

When an arteritic origin is excluded, many authors recommend that patients are immediately transferred to a neurology department or stroke unit [1,2,3] because CRAO and BRAO are associated with an increased risk of cerebral stroke (RAOs and cerebral strokes share the same vascular risk factors) [60]. Roslak-Walek et al. [60] investigated whether the risks for cardiovascular events and cerebral strokes differ between CRAO and BRAO, but could not find a statistically significant difference. Within a follow-up-period of 11 years, Roslak-Walek et al. reported that about 10% of patients with RAO experienced an ischemic cerebral stroke, 2.3% suffered a myocardial infarction and the overall mortality rate was 23% [60].

Neurological examinations: To rule out orbital or intracranial pathologies, CT or cranial magnetic resonance imaging (MRI) can be initiated [61]. Although 90% of patients did not have neurological symptoms, Lauda et al. found, in a retrospective MRI study of patients with CRAO (100 patients), BRAO (45 patients) and amaurosis fugax (68 patients), that 23% exhibited brain infarctions [62]. Callizo et al. found cerebral strokes in 14% of 77 CRAO patients, with five of those occurring within one month after the CRAO [15]. 

MRI can provide information about concurrent cerebral ischemia in patients without accompanying neurological symptoms. Magnetic resonance angiography (MRA) of the head and neck may be more accurate in detecting vascular occlusive diseases. Carotid artery dissection may require CT or computed tomography angiography (CT/CTA) or MRI/MRA of the neck [63].

Although there is controversy regarding the need for a complete neurological examination including CT or MRI in RAO patients without neurological symptoms, there is an agreement that an urgent search for risk factors and their elimination has high priority to prevent further medical events [15,60,64].

## 5. Treatment of Acute Retinal Artery Occlusion

Because RAO causes irreversible visual loss, it should be treated as an emergency. Within the first 24 h, several treatment options can be considered. However, the patient should be informed that these therapies are controversial and cannot guarantee a benefit [1]. Data in the literature suggest that the maximum retinal tolerance time for effective reperfusion is up to 6 h after CRAO [65,66] and that only a rare subgroup of patients have viable tissue after a longer period [66].

Laying down in a supine position might improve ocular perfusion. This positioning is easy to perform, non-invasive, safe and has no side effects. Ocular massage is recommended: pressure is applied for 10–15 s and then released, and this procedure is continued for 3–5 min. Ocular massage causes retinal arterial dilatation and fluctuations in the intraocular pressure (IOP) [67], leading to changes in the arterial blood flow that may mobilize the embolus [1,2].

Another option to re-establish perfusion of an occluded vessel is to decrease the intraocular pressure. The rationale is that a decreased intraocular pressure facilitates, or increases, the arterial perfusion, which may mobilize the embolus, so that it migrates to a more peripheral part of the vessel [1,67,68]. The intraocular pressure may also be lowered by administering 1% topical apraclonidine (topical sympathomimetic agents), 0.5% timolol (topical beta-adrenergic blocking agents) and 500 mg of intravenous acetazolamide [1,69]. Hyperosmotic agents such as Mannitol and glycerol can also be considered to decrease the IOP more rapidly [1].

Anterior chamber paracentesis, which represents the quickest procedure to lower the eye pressure, is controversial, because the procedure has risk factors such as intraocular hypotonia or endophthalmitis. Atebara et al. compared patients treated with inhalative carbogen and paracentesis with untreated controls, but found only a small benefit of this therapeutical approach [70]. Similarly, Fieß et al. compared patients with CRAO treated with and without anterior chamber paracentesis, and did not find a significant difference between these treatment options [71]. Other studies in patients treated with ocular massage, anterior chamber paracentesis and/or hemodilution did not reveal significant positive therapeutical effects compared with untreated control groups [72,73].

Other options to induce vasodilatation are treatment with sublingual isosorbide dinitrate or “rebreathing” into a paper bag to elevate blood carbon dioxide concentration. This activity causes respiratory acidosis, resulting in vasodilatation [1]. The inhalation of carbogen has also been mentioned as a therapeutical approach [1,72,74]. This was administered as a combination of 5% carbon dioxide and 95% oxygen, which is thought to promote vasodilatation of the arterioles via carbon dioxide and to improve the oxygenation of the ischemic retina through the high concentration of inspired oxygen. However, this effect remains equivocal, because vasodilatation could not be shown for this therapy in healthy volunteers [6,74]. To identify the optimal ratio between carbon dioxide and oxygen for the treatment of CRAO, Schmetter et al. investigated the pulsatile ocular blood flow in 20 healthy individuals by means of laser-interferometric measurement. An inhalation of 5% carbon dioxide and 95% oxygen was the most satisfactory ratio to maintain, or even increase, the pulsatile ocular blood flow in the macula and the optic disc [75].

The treatment of CRAO with hyperbaric oxygen revealed promising results. When applied within 8 h of the onset of the symptoms, Hanley et al. described an 83% chance of visual improvement [76]. The rationale is to increase the oxygen level of the blood and to provide oxygen to the retina via diffusion from the choroidal circulation [76,77,78,79,80]. A meta-analysis revealed that hyperbaric oxygen therapy appears beneficial [79], but in a retrospective study, Rosignoli et al. could not verify such an effect [80].

If the occluding embolus is visible, transluminal YAG-Laser embolysis can be considered [81,82,83]. This procedure should be performed within 6 h after the onset of the symptoms. Shots of 0.5 mJ (milli-Joule) were applied directly to the embolus in order to eject it into the vitreous via an opening in the arteriole. However, this procedure comes with risks of severe complications such as retinal or vitreous hemorrhage, retinal breaks, choroidal neo-vascularization and the formation of epiretinal membranes [1,81,82,83].

Thrombolysis has also been used to treat both CRAO and BRAO. Schrag et al. found that fibrinolysis might be beneficial when given early, ideally within 4.5 h after the onset of symptoms [73]. The thrombolytic agents can be delivered either directly into the ophthalmic artery via selective catheterization from the femoral artery, or peripherally through an intravenous infusion. However, Schumacher et al. compared local intra-arterial fibrinolysis using clot-busting tPA with a conservative treatment and did not show a significant difference between the two treatment groups [84]; furthermore, symptomatic intracranial hemorrhage occurred in some patients and the investigators concluded that this therapy would not be recommended [65]. Another study compared intravenous tPA to placebo given within 6 h in CRAO patients. After one week, 25% of the patients in the tPA group showed a visual improvement of at least three lines, whereas no visual benefit was found in the placebo group. However, there was no beneficial long-term effect for visual acuity, indicating that re-occlusion is a potential problem. In addition, symptomatic intracranial hemorrhage was also described [65].

## 6. Conclusions

Retinal artery occlusions often lead to severe visual loss. The incidence is estimated to range between 0.86 and 2 persons per 100,000, and increases with age. Most patients have vascular risk factors. Recommended examinations include ECG, duplex ultrasound investigation of the carotid artery and the cervical arteries, and blood tests. Since there is strong evidence that RAOs are associated with cardiovascular and other diseases, interdisciplinary cooperation is strongly recommended to prevent other severe pathologies in patients with RAO. Although there are several therapeutical approaches, an unequivocal therapeutic approach is still not available. Since the therapeutical options are not satisfactory, a thorough evaluation of the risk factors, followed by an immediate initiation of appropriate therapies, is important to reduce the risk of further ocular events, cardiovascular disease, stroke and mortality.

## Data Availability

No new data were created or analyzed in this study. Data sharing is not applicable to this article.
